# Sensitivity of 2-Hour Gastric Emptying Scintigraphy for Delayed Gastric Emptying Confirmed at 4 Hours: A Retrospective Single-Center Study

**DOI:** 10.3390/diagnostics16213683

**Published:** 2026-11-07

**Authors:** Linda Atencio Ibarra, Raul Cañadas Garrido, Carlos Granados, Mayumi Tacuri, Grecia Claussen, Martin Alonso Rondón Sepúlveda, Katy Elena Retamoza Chamorro, Elkin Navarro Quiroz, Roberto Navarro Quiroz, Nataly Solano Llanos, Alcides Manuel Rodríguez Mercado, Albis Hani de Ardila

**Affiliations:** 1 Facultad de Medicina, Pontificia Universidad Javeriana, Bogotá 110231, Colombia; lindatencioi@gmail.com (L.A.I.); rcanadas@javeriana.edu.co (R.C.G.); cegranados@husi.org.co (C.G.); mayumi.tacuri@upch.pe (M.T.); gacpf2017@gmail.com (G.C.); martin.rondon@javeriana.edu.co (M.A.R.S.); albishani@gmail.com (A.H.d.A.); 2 Department of Internal Medicine, Hospital Universitario San Ignacio, Bogotá 110231, Colombia; 3 Gastroenterology Unit, Department of Internal Medicine, Hospital Universitario San Ignacio, Bogotá 110231, Colombia; 4 Facultad de Medicina, Universidad Peruana Cayetano Heredia, Lima 15102, Peru; 5 Facultad de Medicina, Universidad Nacional Federico Villarreal, Lima 15001, Peru; 6 Department of Clinical Epidemiology and Biostatistics, Pontificia Universidad Javeriana, Bogotá 110231, Colombia; 7 Departamento de Ciencias Naturales y Exactas, Universidad de la Costa—CUC, Barranquilla 080002, Colombia; kretamoz2@cuc.edu.co; 8 Centro de Investigaciones en Ciencias de la Vida (CICV), Facultad de Ciencias Básicas y Biomédicas, Universidad Simón Bolívar, Barranquilla 080002, Colombia; alrodriguezm89@gmail.com; 9 Facultad de Ingeniería, Tecnológico de Antioquia Institución Universitaria, Medellín 050034, Colombia; roberto.navarro@tdea.edu.co or robertcnavarro@gmail.com; 10 Centro de Investigaciones en Ciencias de la Vida (CICV), Facultad de Ciencias de la Salud, Universidad Simón Bolívar, Barranquilla 080002, Colombia; nataly.solano@unisimon.edu.co

**Keywords:** gastroparesis, gastric-emptying scintigraphy, abbreviated protocol, diagnostic sensitivity, diabetes mellitus, nuclear medicine

## Abstract

**Background/Objectives:** Abbreviated gastric-emptying scintigraphy (GES) protocols could save resources. We estimated how often the 2 h criterion (retention ≥ 60%) identifies patients whose delayed gastric emptying is confirmed at 4 h (retention ≥ 10%). **Methods:** This is a retrospective single-center study of adults with 4 h-confirmed delayed gastric emptying undergoing standardized GES at a tertiary hospital in Bogotá, Colombia (2010–2024). Sensitivity with Wilson 95% confidence intervals (CIs) was estimated overall and in exploratory subgroups. **Results:** Of 101 records, 97 adults were analyzed (78.4% women; 18.6% with diabetes). The median 4 h retention was 20.0% (IQR 15.0–27.0%). The 2 h criterion identified 41/97 patients (sensitivity 42.3%; 95% CI 32.9–52.2%); 56 (57.7%) would have been missed at 2 h. Sensitivity was 30.0%, 36.7% and 87.5% for mild, moderate and severe delay, respectively. It was 27.8% in patients with diabetes and 45.6% in those without (*p* = 0.196; underpowered comparison). Because no patient had normal 4 h retention, the specificity and predictive values could not be estimated. **Conclusions:** A 2 h retention below 60% does not exclude delayed gastric emptying, particularly when the delay is mild or moderate; the 4 h acquisition should be completed in these patients. Prospective studies that include patients with normal emptying are needed.

## 1. Introduction

Gastroparesis is a chronic disorder defined by the delayed gastric emptying of solids in the absence of mechanical obstruction [1,2]. Its clinical picture includes nausea, vomiting, early satiety, postprandial fullness, bloating, upper abdominal pain and, in advanced cases, weight loss, with a substantial impact on quality of life and nutritional status [1,2].

Diabetes mellitus is a major cause of gastroparesis, in which autonomic neuropathy and the loss of the interstitial cells of Cajal contribute to impaired antral contractility and antropyloric coordination [3,4,5]. Idiopathic and post-surgical forms are also frequent, which broadens the clinical relevance of an accurate and timely diagnosis [2,3].

The diagnosis requires an objective demonstration of delayed gastric emptying [2,6]. Solid-meal GES is the reference method, and the joint consensus of the American Neurogastroenterology and Motility Society (ANMS) and the Society of Nuclear Medicine (now SNMMI) standardized a low-fat, egg-white meal with imaging at 0, 1, 2 and 4 h; retention > 60% at 2 h and/or >10% at 4 h is considered delayed [7,8,9].

The 4 h acquisition, however, occupies the gamma camera and the patient for a prolonged period, and several groups have explored shortened protocols [10,11]. Ziessman et al. showed that, compared with 2 h, extending imaging to 4 h increased the number of abnormal studies by 29%, and the 2 h criterion had a sensitivity of 59% relative to 4 h [12]; Pathikonda et al. likewise found that 4 h imaging identified additional patients with delayed emptying who were not detected at 2 h [13]. Bonta et al. proposed terminating the study at 2 h only when retention was clearly normal or clearly abnormal [10], and a multicenter validation of this approach (2 h retention < 45% considered normal, >65% abnormal, intermediate values imaged to 4 h) allowed early termination in 74.9% of studies with a sensitivity of 92.4% and a specificity of 96.9% [11].

The data from Latin America are scarce. In this study, we estimated the sensitivity of the 2 h criterion (retention ≥ 60%) for delayed gastric emptying confirmed at 4 h (retention ≥ 10%) in adults evaluated at a tertiary referral center in Colombia, and explored clinical and temporal factors associated with a missed diagnosis at 2 h.

## 2. Materials and Methods

### 2.1. Study Design, Setting and Participants

We performed a retrospective, single-center diagnostic study at Hospital Universitario San Ignacio, Bogotá, Colombia, covering GES studies performed between January 2010 and December 2024. Reporting follows the STARD 2015 guidelines as far as applicable [14]. The study database included only adults whose 4 h gastric retention met the reference criterion for delayed gastric emptying (≥10%); studies with normal 4 h retention were not included. Consequently, all analyzed patients were reference-standard positive.

Inclusion criteria were age ≥ 18 years, a complete GES study with 2 h and 4 h acquisitions, and 4 h retention ≥ 10%. Exclusion criteria were pregnancy or lactation, prior gastric surgery, known mechanical gastric outlet obstruction and inability to complete the 4 h study. When a patient had more than one study, only the first was analyzed. Age, sex, diabetes mellitus, nausea and/or vomiting, other symptoms and comorbidities were extracted from the clinical records.

### 2.2. Gastric-Emptying Scintigraphy Protocol

GES was performed according to the ANMS/SNMMI consensus recommendations [7,8,15,16]. After an overnight fast (minimum 6 h), patients ingested the consensus low-fat liquid egg-white meal (with two slices of white bread, jam and water; approximately 255 kcal: 72% carbohydrate, 24% protein, 2% fat and 2% fiber), labeled with 37 MBq (1 mCi) of ^99m^Tc-sulfur colloid and consumed within 10 min. Prokinetics, opioids and anticholinergic agents, as well as other drugs known to affect gastric motility, including glucagon-like peptide-1 receptor agonists (GLP-1 RAs), were withheld for 48–72 h before the study. Fasting blood glucose was measured before meal ingestion, and the study proceeded only if it was <275 mg/dL, as recommended by the consensus [7]. Patients with diabetes took half of their usual insulin dose with the test meal, and oral hypoglycemic agents were withheld on the morning of the study.

Anterior and posterior images of the upper abdomen (1 min per view) were acquired immediately after meal ingestion and at 1, 2 and 4 h, using a dual-head gamma camera with low-energy high-resolution collimators; the camera was replaced in 2018 by a Philips dual-head SPECT system (Philips Healthcare, Best, The Netherlands). Meal preparation and the acquisition protocol remained standardized and unchanged throughout the 2010–2024 study period. An additional 3 h image was acquired in a subset of patients at the discretion of the referring physician to better characterize late-phase emptying. Gastric counts were obtained from regions of interest, the geometric mean of anterior and posterior counts was calculated and corrected for ^99m^Tc decay, and results were expressed as percent gastric retention relative to time 0.

### 2.3. Index Test, Reference Standard and Definitions

The index test was 2 h retention ≥ 60% and the reference standard was 4 h retention ≥ 10%, the thresholds used in routine reporting at our institution and derived from the consensus normal values (upper limits of 60% at 2 h and 10% at 4 h) [7,8]; because the consensus defines delay as retention above these limits, a sensitivity analysis restricted to patients with 4 h retention > 10% was also performed. Severity of delayed emptying was graded according to 4 h retention as mild (10–20%), moderate (21–35%), severe (36–50%) or very severe (>50%) [7]. As an exploratory analysis, 3 h retention ≥ 30% was evaluated in patients with a 3 h image, using the threshold cited in the consensus document [7]. Both index and reference values were derived from the same acquisition and were obtained as part of routine clinical reporting.

### 2.4. Data Management

In part of the source database (65 of 101 records), retention values had been entered as fractions (e.g., 0.55) rather than percentages (e.g., 55%). All values were harmonized to percentages before analysis and verified against the original scintigraphy reports. Patients were not identifiable in the analysis dataset.

### 2.5. Statistical Analysis

Continuous variables are presented as mean ± standard deviation or median (interquartile range, IQR) and categorical variables as *n* (%). Sensitivity was calculated as the proportion of 4 h-positive patients with 2 h retention ≥ 60%, with Wilson score 95% CIs [17]. Because every analyzed patient met the reference standard, specificity, positive and negative predictive values, likelihood ratios and receiver operating characteristic (ROC) analysis could not be estimated; this design is susceptible to spectrum effects, and sensitivity estimates may not transfer to unselected referral populations [18,19].

Sensitivity was compared across exploratory subgroups (diabetes, nausea/vomiting, sex, study period [2010–2017 vs. 2018–2024] and 4 h severity grade) using Fisher’s exact test; for diabetes, the difference in sensitivity was reported with a Newcombe 95% CI [20] and post hoc power was calculated. Temporal trends were assessed with Spearman’s correlation between calendar year and 2 h detection or 4 h retention. Correlations between 2, 3 and 4 h retention were assessed with Spearman’s ρ, and 4 h retention was compared between groups with the Mann–Whitney U test. Sensitivity across alternative 2 h thresholds (35–70%) was described as an exploratory analysis. A sensitivity analysis included all 101 records. No formal a priori sample size calculation was performed; with 97 patients, the 95% CI half-width for a sensitivity of approximately 40% is about ±10 percentage points. Tests were two-sided with α = 0.05, without adjustment for multiple comparisons; subgroup findings are hypothesis-generating. Analyses were performed in Python 3.10 (pandas (version 2.0.3), SciPy (version 1.10.1), Matplotlib (version 3.7.2)); the analysis code is provided as Supplementary Materials.

## 3. Results

### 3.1. Participants

**Figure 1:**
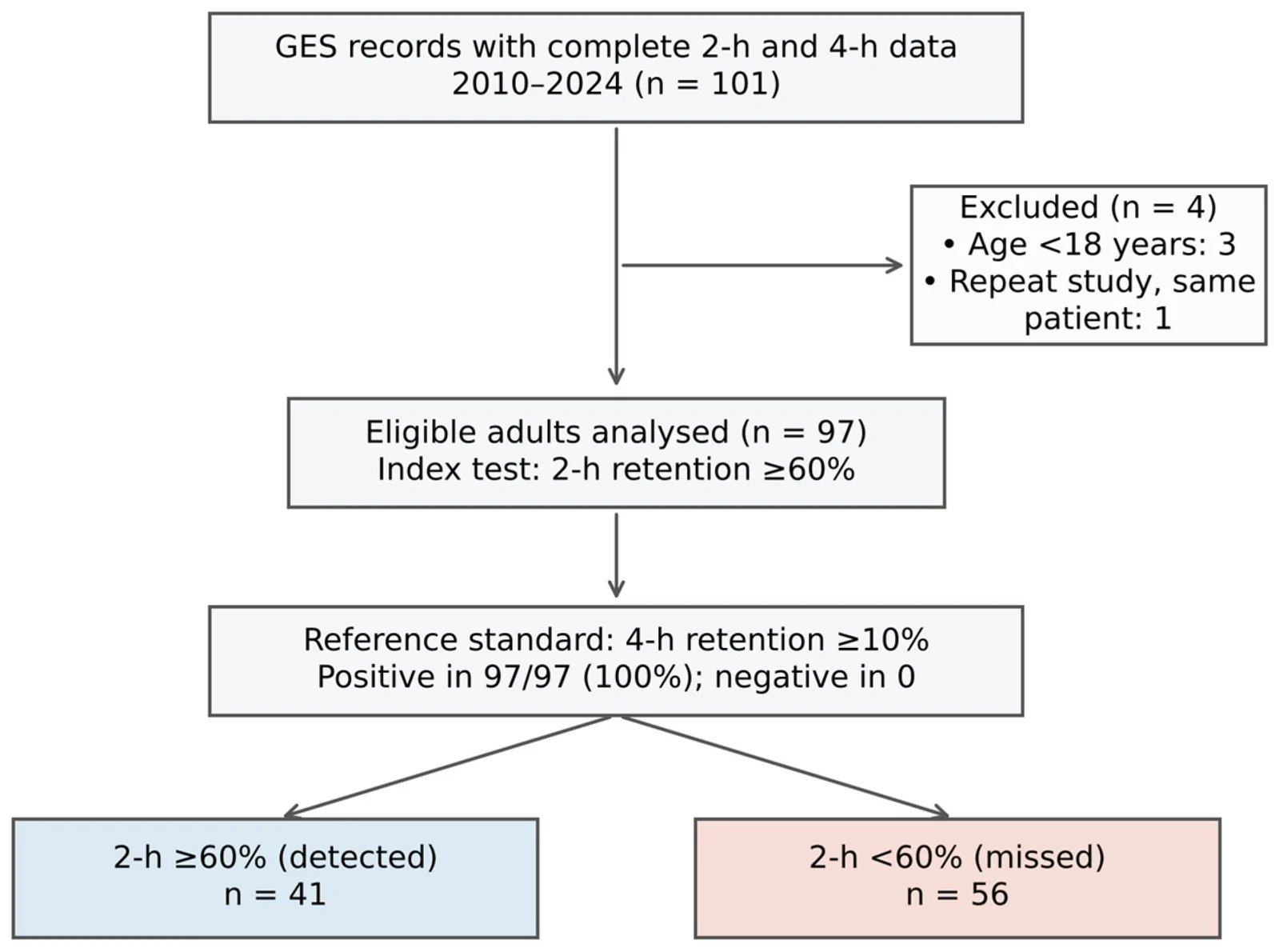
Flow of participants (STARD diagram). Blue box indicates patients detected at 2 h (retention ≥ 60%); red box indicates patients missed at 2 h (retention < 60%). All analyzed patients had 4 h retention ≥ 10% (reference-standard positive); no reference-standard-negative patients were available.

Of 101 GES records, three patients younger than 18 years and one repeat study of the same patient were excluded, leaving 97 adults for analysis (Figure 1). The mean age was 49.9 ± 17.5 years and 76 (78.4%) were women; 18 (18.6%) had diabetes mellitus and 45 (46.4%) reported nausea and/or vomiting (Table 1). The number of eligible patients per year ranged from 3 to 12.

### 3.2. Gastric Retention Values

**Figure 2:**
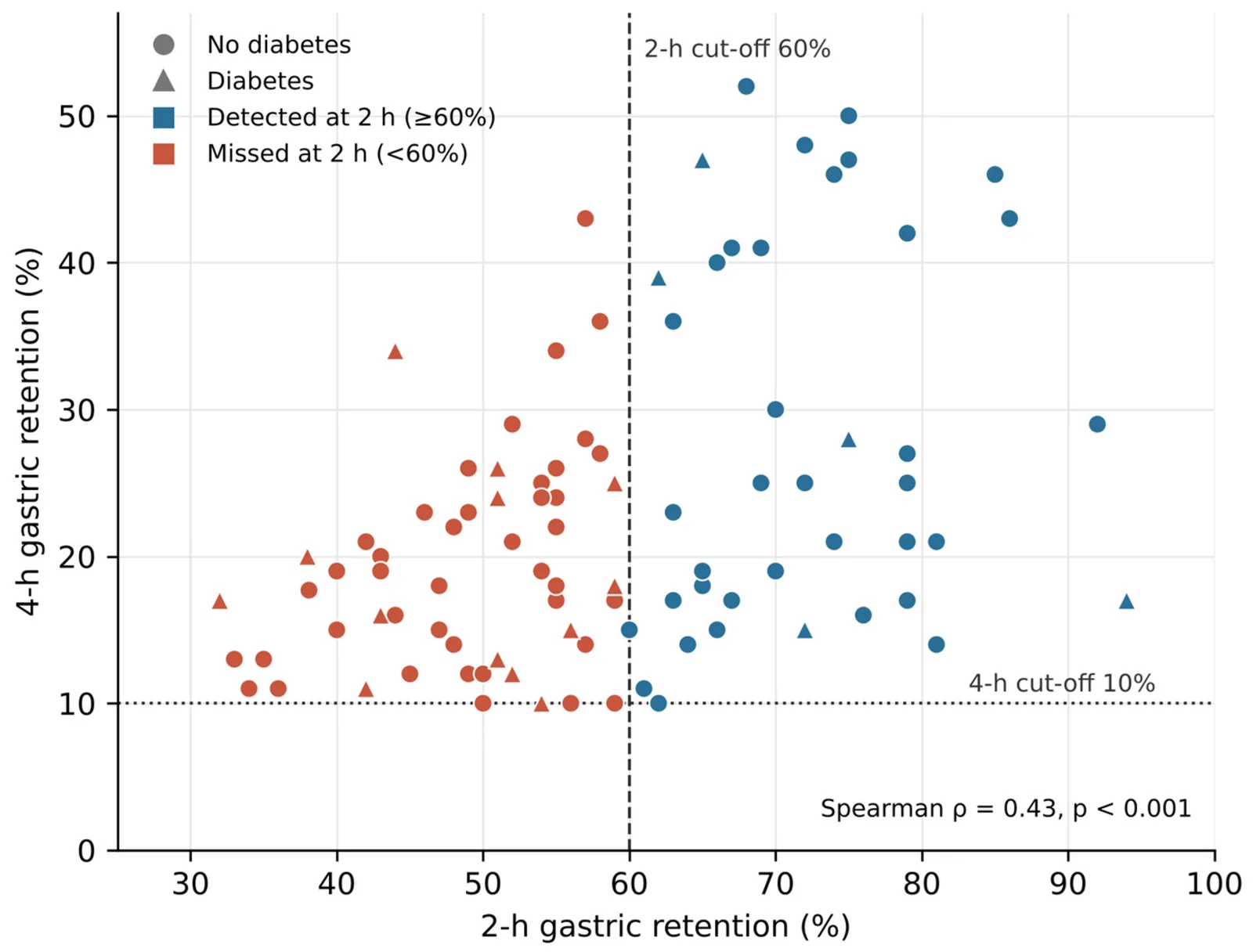
Relationship between 2 h and 4 h gastric retention (*n* = 97). Blue: 2 h retention ≥ 60% (detected); red: 2 h retention < 60% (missed). Circles, patients without diabetes; triangles, patients with diabetes. Dashed line, 2 h threshold (60%); dotted line, 4 h threshold (10%).

The median retention was 57.0% (IQR 49.0–68.0%) at 2 h and 20.0% (IQR 15.0–27.0%) at 4 h (Table 2). According to 4 h retention, delayed emptying was mild in 50 (51.5%), moderate in 30 (30.9%), severe in 16 (16.5%) and very severe in 1 (1.0%) patients. Retention at 2 h correlated moderately with retention at 4 h (ρ = 0.43, *p* < 0.001; Figure 2), whereas, in the 61 patients with a 3 h image, 3 h retention correlated more strongly with 4 h retention (ρ = 0.76, *p* < 0.001).

### 3.3. Sensitivity of the 2 h Criterion

The 2 h criterion identified 41 of 97 patients with delayed emptying at 4 h (sensitivity 42.3%; 95% CI 32.9–52.2%), whereas 56 patients (57.7%) had 2 h retention < 60% (Table 3). Among the missed patients, the median 2 h retention was 50.0% (IQR 43.0–55.0%), and their 4 h retention was lower than that of the detected patients (median 18.0% vs. 25.0%; *p* < 0.001). Restricting the analysis to the 92 patients with 4 h retention > 10% (excluding 5 with exactly 10%), sensitivity was 43.5% (40/92; 95% CI 33.8–53.7%). Including all 101 records, sensitivity was 42.6% (43/101; 95% CI 33.4–52.3%).

### 3.4. Exploratory Subgroup Analyses

**Figure 3:**
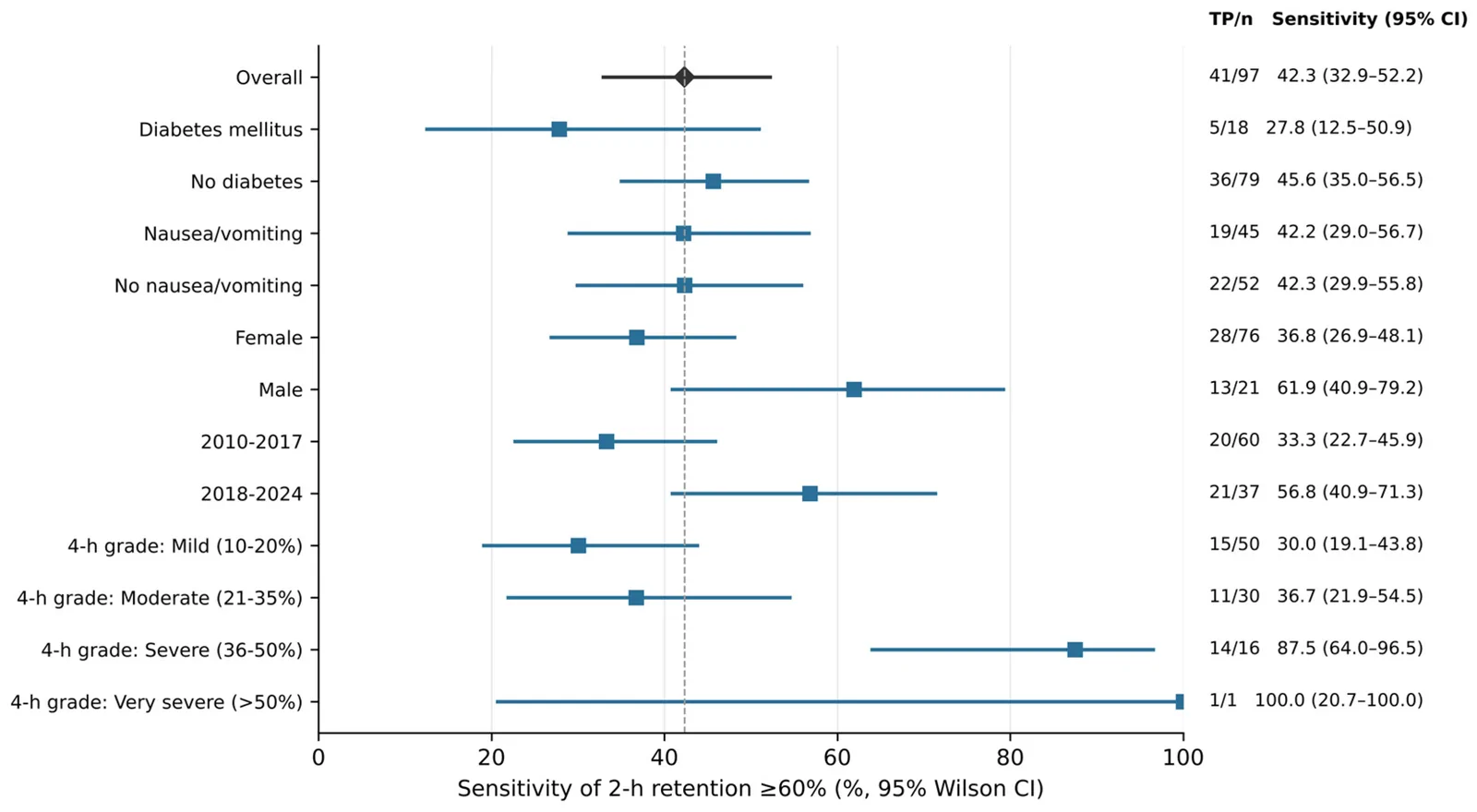
Sensitivity of 2 h retention ≥ 60% overall and by subgroup, with Wilson 95% CIs. The dashed line marks the overall estimate.

Sensitivity increased with the severity of 4 h retention (30.0% in mild, 36.7% in moderate and 87.5% in severe delay; ρ = 0.35, *p* < 0.001) (Table 4, Figure 3). Sensitivity was 27.8% (5/18; 95% CI 12.5–50.9%) in patients with diabetes and 45.6% (36/79; 95% CI 35.0–56.5%) in those without; the difference (−17.8 percentage points; 95% CI −36.6 to 7.6) was not statistically significant (*p* = 0.196), and the post hoc power to detect it was 27%. Detecting a 20-percentage-point difference (e.g., 45% vs. 25%) with 80% power would require approximately 89 patients per group. Sensitivity did not differ by nausea/vomiting (*p* = 1.000). It was higher in men than in women (*p* = 0.048) and in 2018–2024 than in 2010–2017 (56.8% vs. 33.3%; *p* = 0.034), although no significant linear trend with calendar year was observed for 2 h detection (ρ = 0.17, *p* = 0.099) or for 4 h retention (ρ = 0.14, *p* = 0.167).

### 3.5. Alternative 2 h Thresholds and 3 h Retention

**Figure 4:**
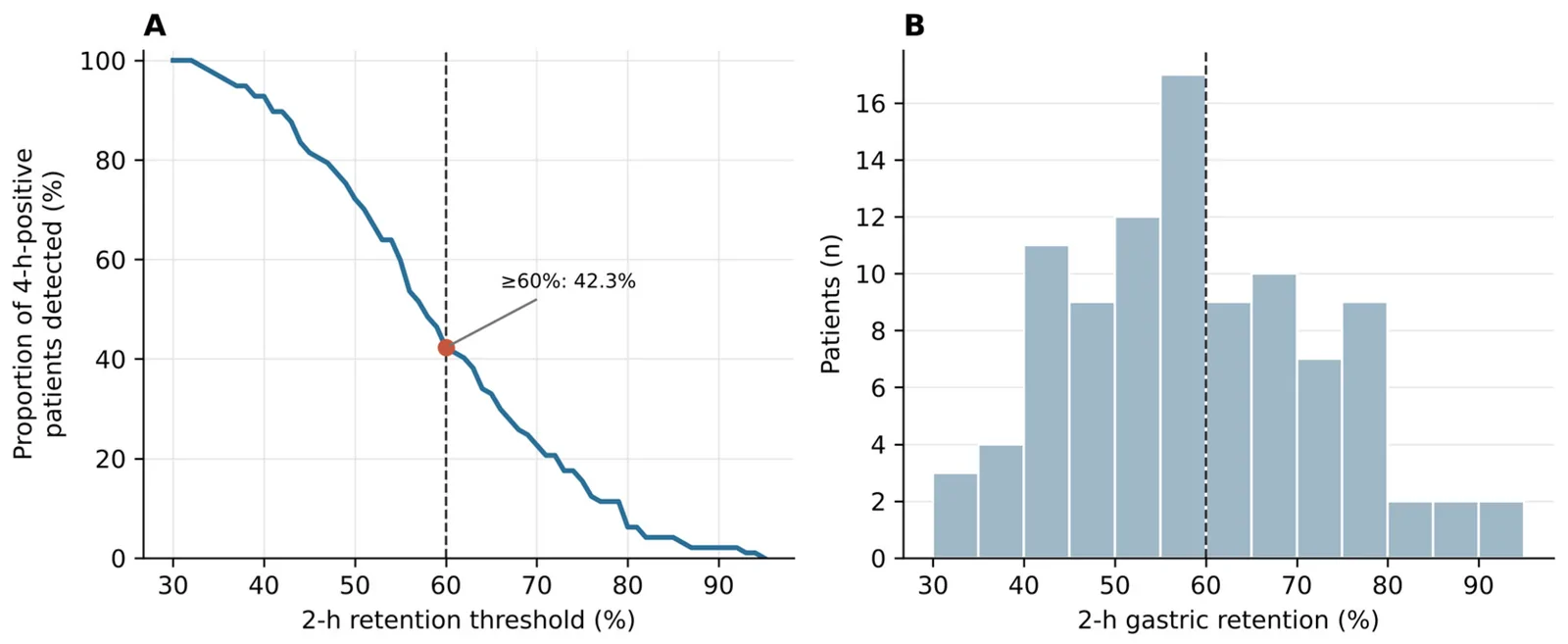
(**A**) Proportion of patients with 4 h-confirmed delayed emptying identified across 2 h retention thresholds; the red point marks the consensus threshold (≥60%). (**B**) Distribution of 2 h retention; the dashed line marks 60%.

**Figure 5:**
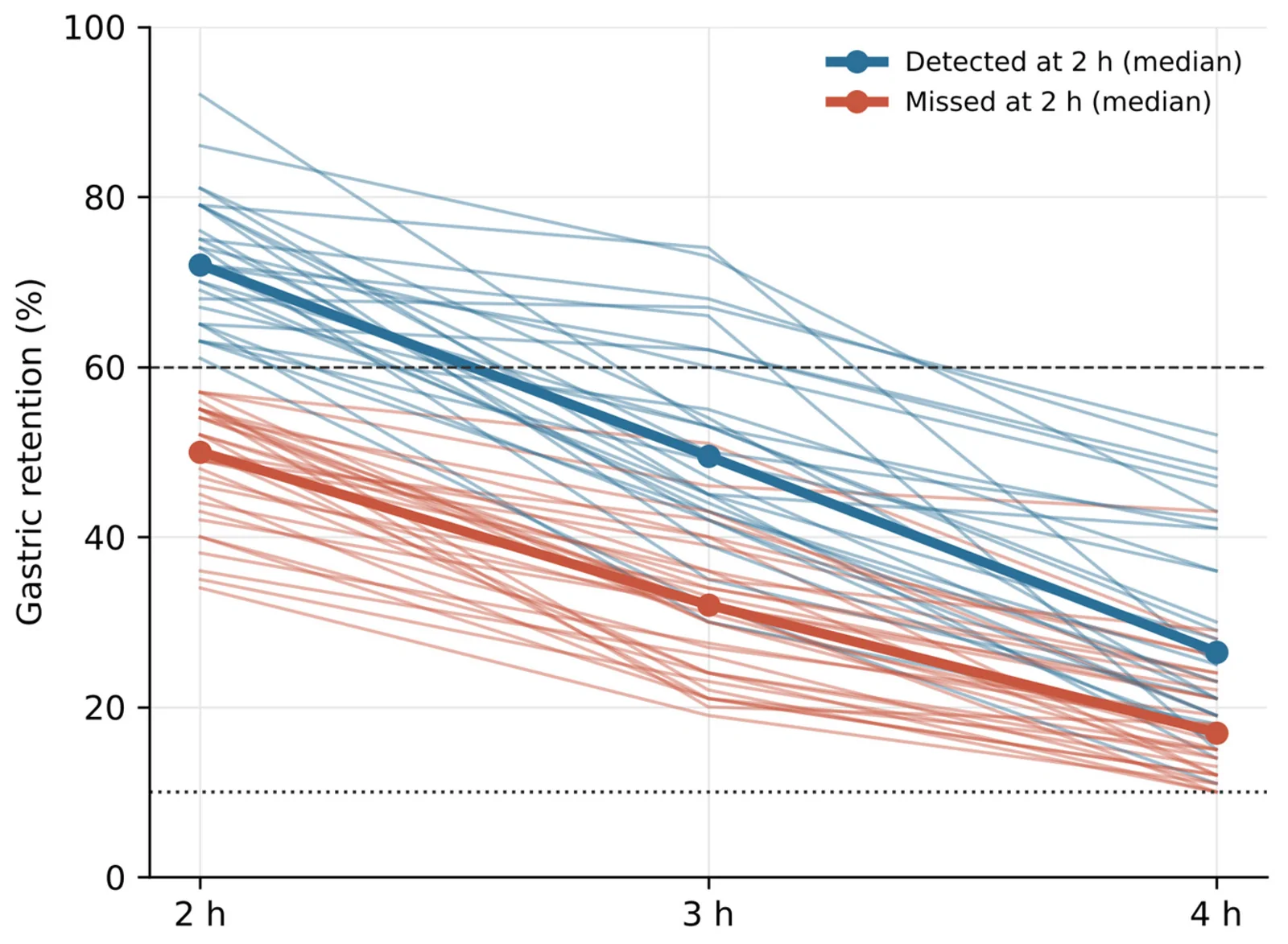
Individual retention trajectories at 2, 3 and 4 h in patients with a 3 h image (*n* = 61). Thick lines show medians for patients detected (blue) and missed (red) by the 2 h criterion. Dashed line, 60%; dotted line, 10%.

Lowering the 2 h threshold increased the proportion of 4 h-positive patients identified (Table 5, Figure 4): 59.8% at ≥55%, 72.2% at ≥50% and 92.8% at ≥40%. These figures describe sensitivity only; because 2 h values between 30% and 60% are within the normal range [7,8], the corresponding false-positive rates cannot be assessed in this cohort. In the 61 patients with a 3 h image, 3 h retention ≥ 30% identified 48 (78.7%; 95% CI 66.9–87.1%), compared with 28 (45.9%; 95% CI 34.0–58.3%) for the 2 h criterion in the same patients; all patients detected at 2 h were also above the 3 h threshold. Individual emptying trajectories are shown in Figure 5.

### 3.6. Symptoms

Four-hour retention did not differ between patients with and without nausea and/or vomiting (median 19.0% vs. 20.5%; *p* = 0.839) or between patients with and without diabetes (median 17.5% vs. 21.0%; *p* = 0.455).

## 4. Discussion

In this retrospective series of 97 adults with delayed gastric emptying confirmed at 4 h, the 2 h criterion of ≥60% retention identified only 42.3% of patients; 56 patients (57.7%) would have been classified as normal had the study been stopped at 2 h. Missed cases were concentrated among patients with mild or moderate delay, whereas most patients with severe delay were identified at 2 h. These findings support completing the 4 h acquisition whenever 2 h retention is below 60%.

Our estimate is lower than the 59% reported by Ziessman et al. [12] and is consistent with the observation that 4 h imaging detects patients missed at 2 h [13]. Sensitivity is not a fixed property of a test: it depends on the spectrum of disease in the population studied [18,19]. In our cohort, more than half of the patients had mild delay (10–20% at 4 h), a group in which the 2 h criterion performed poorly, which probably explains much of the difference. Conversely, the multicenter validation by Pelletier-Galarneau et al. achieved high sensitivity because patients with intermediate 2 h values (45–65%) continued to 4 h [10,11]; such a strategy preserves diagnostic accuracy by not relying on the 2 h value alone.

Our data do not allow us to assess specificity, predictive values or ROC-based thresholds, because no patient with normal 4 h retention was included. In line with the consensus criteria, a 2 h retention > 60% is itself abnormal [7], and, in this cohort, every such patient also had delayed emptying at 4 h; however, without reference-negative patients, the false-positive rate of this criterion in an unselected population cannot be quantified here.

From a practical perspective, the potential benefit of a shortened protocol is limited. Within this cohort, 42.3% of studies could have been terminated at 2 h with a positive result; in an unselected referral population, every patient with 2 h retention < 60%—including those with normal emptying—would still require 4 h imaging, so the proportion of studies terminated early would be lower. Given the volume of this series (on average, 6.5 eligible patients per year), the reduction in gamma-camera time at our center would be modest, and any gain must be weighed against the risk of missing mild and moderate gastroparesis.

The lower point estimate of sensitivity in patients with diabetes (27.8% vs. 45.6%) should be interpreted with caution. With only 18 patients with diabetes, the confidence interval is wide, the difference was not statistically significant and the comparison was underpowered. Gastric emptying in diabetes is heterogeneous and is influenced by autonomic neuropathy, acute glycemia and medications, including glucagon-like peptide-1 receptor agonists (GLP-1 RAs) [4,21,22]; although medications were withheld and fasting glucose was checked according to the protocol, individual glucose values and medication histories were not available in our research database for analysis. We therefore cannot draw firm conclusions for this subgroup, and larger studies with standardized recording of glycemia and medication use are required.

Sensitivity was higher in 2018–2024 than in 2010–2017, although no significant linear trend with calendar year was observed. The change in gamma camera in 2018 coincides with this period split; although the acquisition protocol and test meal were unchanged, an effect of the new equipment cannot be excluded, and changes in the characteristics of referred patients or chance may also contribute. This underlines a limitation of a 15-year retrospective series. The higher sensitivity observed in men is likewise exploratory and may be a chance finding given the number of comparisons.

Alternative parameters merit consideration. Lower 2 h thresholds increased sensitivity, but, because the 2 h values of 30–60% fall within the normal range [7,8], they would inevitably classify many patients with normal emptying as abnormal; such thresholds should not be adopted without studies that include reference-negative patients. In the subset with a 3 h image, 3 h retention identified a larger proportion of patients than the 2 h criterion and correlated more strongly with 4 h retention, suggesting that a 3 h acquisition might be a better compromise than a 2 h acquisition; this exploratory observation requires validation. The gastric half-emptying time could not be estimated reliably because 1 h values were not available in the study database.

This study has several limitations. First, it is retrospective and the database included only patients with delayed emptying at 4 h, which precludes the estimation of specificity and predictive values and introduces spectrum bias. Second, the relatively small sample was accrued over 15 years. Although the acquisition protocol, test meal and radiopharmaceutical preparation remained unchanged, the gamma camera was replaced in 2018 and the characteristics of referred patients may have changed over time, which could partly explain the higher sensitivity observed in 2018–2024. Third, although medications affecting gastric motility were withheld and fasting glucose was checked according to the consensus protocol, glucose values and medication histories were not recorded in the research database and could not be analyzed. Fourth, symptoms were obtained from clinical records rather than validated instruments such as the Gastroparesis Cardinal Symptom Index. Fifth, the index and reference values were obtained from the same acquisition and were not interpreted independently. Finally, this is a single-center study, and retention values recorded in different formats had to be harmonized, although all were verified against the original reports. Strengths include the use of a standardized consensus meal and acquisition protocol and transparent reporting of the full distribution of retention values.

## 5. Conclusions

In adults with delayed gastric emptying confirmed at 4 h, a 2 h retention ≥ 60% identified fewer than half of the patients, and most missed patients had mild or moderate delay. A 2 h retention below 60% should not be used to exclude gastroparesis, and the 4 h acquisition should be completed in these patients. Because our cohort did not include patients with normal emptying, these data do not support alternative 2 h thresholds or conclusions about specificity, and the findings in patients with diabetes are inconclusive. Prospective, multicenter studies including unselected referrals, standardized glycemic and medication control, and 3 h imaging are needed before abbreviated protocols can be recommended.

## Figures and Tables

**Table 1:** Characteristics of the study population (*n* = 97).

Characteristic	Value
Age, years, mean ± SD	49.9 ± 17.5
Age, years, median (IQR)	52 (34–64)
Female sex, *n* (%)	76 (78.4)
Diabetes mellitus, *n* (%)	18 (18.6)
Nausea and/or vomiting, *n* (%)	45 (46.4)
Study period 2010–2017, *n* (%)	60 (61.9)
Study period 2018–2024, *n* (%)	37 (38.1)
3 h image available, *n* (%)	61 (62.9)

IQR, interquartile range; SD, standard deviation.

**Table 2:** Gastric retention at 2, 3 and 4 h.

Time	*n*	Mean ± SD, %	Median (IQR), %	Range, %
2 h	97	58.5 ± 14.0	57.0 (49.0–68.0)	32.0–94.0
3 h	61	40.3 ± 14.1	39.0 (30.0–49.0)	19.0–74.0
4 h	97	23.2 ± 10.9	20.0 (15.0–27.0)	10.0–52.0

IQR, interquartile range; SD, standard deviation. The 3 h image was available in a subset of patients.

**Table 3:** Cross-classification of the 2 h index test against the 4 h reference standard (*n* = 97).

2-h Retention	4-h ≥ 10% (Positive)	4-h < 10% (Negative)	Total
≥60% (positive)	41	0	41
<60% (negative)	56	0	56
Total	97	0	97
Sensitivity (95% CI)	42.3% (32.9–52.2%)		
Specificity, PPV, NPV, LRs, AUC	Not estimable		

AUC, area under the receiver operating characteristic curve; LRs, likelihood ratios; NPV, negative predictive value; PPV, positive predictive value. No reference-standard-negative patients were available.

**Table 4:** Sensitivity of 2 h retention ≥ 60% by subgroup.

Subgroup	TP/*n*	Sensitivity, % (95% CI)	*p*
Overall	41/97	42.3 (32.9–52.2)	
Diabetes mellitus	5/18	27.8 (12.5–50.9)	0.196
No diabetes	36/79	45.6 (35.0–56.5)	
Nausea and/or vomiting	19/45	42.2 (29.0–56.7)	1.000
No nausea/vomiting	22/52	42.3 (29.9–55.8)	
Female	28/76	36.8 (26.9–48.1)	0.048
Male	13/21	61.9 (40.9–79.2)	
2010–2017	20/60	33.3 (22.7–45.9)	0.034
2018–2024	21/37	56.8 (40.9–71.3)	
4 h grade: mild (10–20%)	15/50	30.0 (19.1–43.8)	<0.001 ^a^
4 h grade: moderate (21–35%)	11/30	36.7 (21.9–54.5)	
4 h grade: severe (36–50%)	14/16	87.5 (64.0–96.5)	
4 h grade: very severe (>50%)	1/1	100.0 (20.7–100.0)	

CI, confidence interval (Wilson); TP, true positives. *p* values from Fisher’s exact test; ^a^ Spearman correlation between severity grade and 2 h detection. All subgroup analyses are exploratory and unadjusted for multiplicity.

**Table 5:** Proportion of 4 h-positive patients identified at alternative 2 h thresholds (exploratory).

2-h Threshold	Detected, *n*	Sensitivity, %	95% CI
≥70%	22	22.7	15.5–32.0
≥65%	32	33.0	24.4–42.8
≥60%	41	42.3	32.9–52.2
≥55%	58	59.8	49.8–69.0
≥50%	70	72.2	62.5–80.1
≥45%	79	81.4	72.6–87.9
≥40%	90	92.8	85.8–96.5
≥35%	94	96.9	91.3–98.9

CI, confidence interval (Wilson). Specificity cannot be estimated because all patients were reference-standard positive; the ≥60% threshold is the consensus criterion.

## Data Availability

The de-identified data supporting the findings of this study are available from the corresponding author upon reasonable request, subject to institutional data-sharing agreements; they are not publicly available to protect patient confidentiality. The analysis code is provided as Supplementary Materials.

## Supplementary Materials

The following supporting information can be downloaded at: https://www.mdpi.com/article/10.3390/diagnostics16213683/s1, Code S1: Python analysis script (analisisdatosV3.py).

## Abbreviations

The following abbreviations are used in this manuscript:

ANMS	American Neurogastroenterology and Motility Society
AUC	Area under the curve
CI	Confidence interval
GES	Gastric-emptying scintigraphy
GLP-1 RA	Glucagon-like peptide-1 receptor agonist
IQR	Interquartile range
NPV	Negative predictive value
PPV	Positive predictive value
ROC	Receiver operating characteristic
SNMMI	Society of Nuclear Medicine and Molecular Imaging
STARD	Standards for Reporting of Diagnostic Accuracy Studies
